# Supplementary material for: Systemic Sclerosis in Kazakh Patients: A Preliminary Case–Control Immunogenetic Profiling Study
Source: Pathophysiology. 2025 Oct 28;32(4):57. doi: 10.3390/pathophysiology32040057 (PMC12641709; doi:10.3390/pathophysiology32040057)
Supplement: Supplementary file 1 [file pathophysiology-32-00057-s001.zip › Supplementary Table S1.pdf]

SUPPLEMENTARY MATERIAL

Systemic Sclerosis in Kazakh Patients: A Preliminary Case-–Control Immunogenetic Profiling Study

Lina Zaripova*, Abay Baigenzhin, Alyona Boltanova, Zhanna Zhabakova, Maxim Solomadin, Larissa Kozina

JSC National Scientific Medical Center, Astana, Kazakhstan

Supplementary Table S1. Clinical and laboratory characteristics of patients with systemic sclerosis. AC – anti-cell (ICAP, www.anapatterns.org); AITD - autoimmune thyroid disease; ANF – anti-nuclear factor (Anti-cellular antibodies, www.anapatterns.org); EScSG - The European Scleroderma Study Group activity index; f – female; IL-6 – interleukin 6; ILD – interstitial lung disease; LP - likely pathogenic variant; m – male; mRSS – modified Rodnan skin score; P - pathogenic variant; PAH – pulmonary arterial hypertension; RP - Raynaud's phenomenon; SSc - Systemic sclerosis; sSS - secondary Sjogren's syndrome; VUS - variant of uncertain significance.

№	Gender	Diagnosis, organ involvement	mRSS	EScSG	IL-6 (0-10,0 pg/ml)	ANF	Antibodies	ESR	Genetic variants
S1	f	Systemic sclerosis (SSc), chronic course, diffuse form, generalized, activity 1 stage, skin lesions, sclerodactyly, interstitial lung disease (ILD), esophagitis, Raynaud's Phenomenon (RP), cirrhosis	33	2	7,87	1:2560 AC-3	CENP-B	2	LY96(chr8:74922341 CT/C) - variant of uncertain significance (VUS) PTPN22(chr1:114381166 CT/C) –VUS IRAK1(chrX:153278833 GCC/GCCG)-VUS SLC5A11(chr16:24918057 CA/C)-VUS IL6R(chr1:154378136 GC/G) - likely pathogenic variant (LP) TREX1(chr3:48508185 T/TC)-LP
S2	f	SSc, chronic course, diffuse form, generalized, activity 1 stage, skin lesions, RP, esophagitis, polyarthritis, cirrhosis	14	2	0,17	1:640, AC-3, AC-21	CENP-B	12	LY96(chr8:74922341 CT/C)-VUS PTPN22(chr1:114381166 CT/C) –VUS IRAK1(chrX:153278833 GCCCG/GCC)-VUS

S3	f	SSc, subacute course, diffuse form, skin lesions, sclerodactyly, arthralgia, ILD, esophagitis, autoimmune thyroid disease (AITD), RP	9	1,5	1,33	1:320, AC-4/5	SS-A/52	10	LY96(chr8:74922341 CT/C)-VUS<br>PTPN22(chr1:114381166 CT/C) –VUS<br>ITGA2B(chr17:42453072 G/GC (reference sequencing GCC))-LP
S4	f	SSc, chronic course, skin lesions, esophagitis, secondary Sjogren's syndrome (sSS)	2	0,5	0,78	<1:80 AC-0	Negative	7	LY96(chr8:74922341 CT/C)-VUS<br>PTPN22(chr1:114381166 CT/C) –VUS<br>JAZF1(chr7:28220153 C/T)-VUS
S5	f	SSc, chronic course, diffuse form, generalized, activity 2 stage, skin lesions, arthralgia, ILD, esophagitis, AITD, RP, telangiectasia	13	3,5	3,7	1:5120 AC-3	CENP-B	8	LY96(chr8:74922341 CT/C)-VUS<br>PTPN22(chr1:114381166 CT/C) –VUS<br>IRAK1(chrX:153278833 GCC/GCCG)-VUS<br>AIRE(chr21:45711068 TC/T)-LP
S6	f	SSc, chronic course, activity 2, skin lesions, polyarthritis, esophagitis, ILD, PAH, sSS	9	3,5	**21,7**	1:640, AC-4/5, AC-19/20	SS-A/60, U1-snRNP, RNP/Sm, Sm, rib-P0	64	PTPN22(chr1:114381166 CT/C) –VUS<br>IRAK1(chrX:153278833 GCCCG/GCC)-VUS<br>SAMD9L(chr7:92764981 T/TT (reference sequencing TC))-LP<br>SAMD9L(chr7:92761606 GT/G)-LP<br>RBPJ(chr4:26417097GTTTTTTGC/G TTTTTTTG ref GTTTTTTTGC)-VUS<br>IL6R(chr1:154401686 G/A)-LP
S7	f	SSc, chronic course, activity 1, skin lesions, RP	12	3	1,55	1:640, AC-3	CENP-B. SS-A/60 (borderline result)	6	SAMD9L(chr7:92761606 GT/G)-LP<br>REL(chr2:61149099 GT/G)-LP<br>AIRE(chr21:45713024 A/G)-VUS<br>IKZF3(chr17:37922552 T/C)-VUS
S9	f	SSc, chronic course, diffuse form, activity 2, ILD, skin lesions, polyarthralgia, RP	15	5,5	2,33	<1:80 AC-0	Negative	30	PTPN22(chr1:114381166 CT/C) –VUS<br>CLEC16A(chr16:11260320 G/A)-VUS
S10	f	SSc, subacute course, diffuse form, generalized, activity 1,	16	4,5	1,81	1:640,	CENP-B	2	LY96(chr8:74922341 CT/C)-VUS<br>PTPN22(chr1:114381166 CT/C) –VUS

		with lesions of the skin, polyarthralgia, ILD, esophagitis, RP				AC-8/10	(Borderline result)		ITGA2B(chr17:42455791 G/A)-VUS
S11	f	SSc, generalized, stage 2, with lesions of the skin, polyarthralgia, esophagitis, ILD, RP, sSS	14	7	7,14	<1:80 AC-0	Negative	33	LY96(chr8:74922341 CT/C)-VUS PTPN22(chr1:114381166 CT/C) -VUS
S12	f	SSc, generalized, stage 2, with lesions of the skin, joints, ILD, RP, sSS	9	2	6,7	1:320, AC-4/5	SS-A/60, SS-A/52, SS-B. CENP-B (Borderline result)	31	LY96(chr8:74922341 CT/C)-VUS PTPN22(chr1:114381166 CT/C) –VUS AIRE(chr21:45708278 G/C)-VUS
S13	f	SSc, generalized, stage 2, skin lesions, polyarthralgia, RP, ILD, esophagitis	23	5,5	**26,5**	1:320, AC-19/20	SS-A/60, SS-A/52, RNP/Sm, ribP0, U1-snRNP, ds-DNA, Nucleosome Histon	47	PTPRC(chr1:198682102 AT/A)-LP SAMD9L(chr7:92761606 GT/G)-LP
S14	f	SSc, generalized, skin lesions, polyarthralgia, RP, cardiosclerosis, ILD, esophagitis	18	7,5	2,15	1:1280 AC-3, AC-4/5	SS-A/60, SS-A/52, CENP-B	39	LY96(chr8:74922341 CT/C)-VUS PTPN22(chr1:114381166 CT/C) –VUS SLC5A11(chr16:24918057 CA/C)-VUS
S15	f	SSc, chronic course, generalized, skin lesions, RP, esophagitis, arthritis, PAHs, SS, Cushing's syndrome.	26	3,5	4,91	1:5120 AC-3	CENP-B	25	PTPN22(chr1:114381166 CT/C) –VUS IRAK1(chrX:153278833 GCCCG/GCC)-VUS SAMD9L(chr7:92764981 T/TT (reference sequencing TC))-LP SAMD9L(chr7:92761606 GT/G)-LP ABCC2(chr10:101559041 CA/C)-LP AIRE(chr21:45711025 C/G)-VUS

S16	m	Progressive SSc, subacute course, activity 2, skin lesions, ILD, PAH, RP, telangiectasia, esophagitis, arthritis, sSS	15	5,5	3,79	1:320, AC-4, AC-19/20	SS-A/60, SS-A/52, Scl-70	14	LY96(chr8:74922341 CT/C)-VUS<br>PTPN22(chr1:114381166 CT/C) –VUS<br>IKZF3(chr17:37922552 T/C)-VUS
S17	f	SSc, subacute course, diffuse form, activity 2, skin lesions, polyarthralgia, RP	25	3,5	0,34	1:2560 AC-3	CENP-B	12	PTPN22(chr1:114381166 CT/C) –VUS<br>IRAK1(chrX:153278833 GCC/GCCG)-VUS<br>REL(chr2:61149099 GT/G)-LP<br>ABCC2(chr10:101578956 CA/C)-pathogenic variant (P)
S19	f	SSc, subacute course, activity 2, diffuse form activity 2, skin lesions, ILD, PAH, polyarthralgia, heart, RP, sSS	6	1,5	3,88	1:320, AC-4/5	SS-A/60, SS-A/52, U1-sn-RNP, RNP/Sm	41	PTPRC(chr1:198682102 AT/A)-LP<br>CTLA4(chr2:204736165 G/GT)-LP<br>IL6ST(chr5:55265655 G/C)-VUS<br>SAMD9L(chr7:92761606 GT/G)-LP<br>REL(chr2:61149099 GT/G)-LP<br>RBPJ(chr4:26417097 GT/G)-VUS<br>IL18(chr11:112014401 C/T)-VUS
S20	m	SSc, chronic course, diffuse form, generalized, activity 2, skin lesions, polyarthralgia, RP	24	4,5	1,64	1:640, AC-8/10	Negative	12	PTPN22(chr1:114381166 CT/C) –VUS<br>CTLA4(chr2:204736165 G/GT)-LP<br>DNASE1L3(chr3:58183626 G/T)-VUS<br>SFTPD(chr10:81697984 A/G)-VUS
S21	f	SSc, chronic course, activity 1, RP, esophagitis, polyarthralgia, skin lesions, sSS	18	1,5	3,53	1:640, AC-4/5	Sm, U1-snRNP, RNP/Sm,	7	SAMD9L(chr7:92764981 T/TT (reference sequencing TC))-LP<br>CLEC16A(chr16:11260320 G/A)-VUS<br>AFF3(chr2:100623846 GT/G)-LP
S23	f	SSc, stage 2, activity 2, skin lesions, joints, RP, esophagitis	25	5,5	5,26	1:5120 AC-3, AC-4/5	SS-A/60, SS-A/52, CENP-B	63	LY96(chr8:74922341 CT/C)-VUS<br>PTPN22(chr1:114381166 CT/C) –VUS<br>SLC5A11(chr16:24918057 CA/C)-VUS
S24	f	SSc, chronic course, stage 2, activity 1, skin lesions, sclerodactyly, arthralgia, AITD, esophagitis, RP, sSS	22	7,5	8,68	1:640, AC-4/5	SS-A/60, SS-A/52, U1-snRNP, RNP/Sm	46	CLEC16A(chr16:11217782 A/G)-VUS

S25	f	SSc, chronic course, with lesions of the skin, arthralgia, RP, esophagitis	9	0,5	5,26	1:320, AC-19/20, AC-4	SS-A/52	27	LY96(chr8:74922341 CT/C)-VUS<br>RBPJ(chr4:26426085 C/CT)-VUS
S26	f	SSc, chronic course, activity 2, ILD, RP, polyarthralgia, skin lesions	17	6	**14,6**	1:640, AC-4/5	SS-A/52	18	PTPN22(chr1:114381166 CT/C) –VUS<br>PTPRC(chr1:198682102 AT/A)-LP<br>CTLA4(chr2:204732740 GT/G)-LP<br>IL6ST(chr5:55247857 GT/G)-LP<br>PRKCQ(chr10:6527154 AT/A)-VUS<br>PXK(chr3:58385095 T/C)-VUS
S27	f	SSc, generalized, activity 2, skin lesions, arthralgia, esophagitis, ILD, RP, sSS	10	0,5	3,57	1:640, AC-4/5, AC-3	SS-A/52, CENP-B	27	IL6ST(chr5:55265588 AT/A)-LP<br>REL(chr2:61149099 GT/G)-LP<br>TNFAIP3(chr6:138199775 T/TC)-LP<br>IL12B(chr5:158749513 T/C)-VUS
S28	f	SSc, subacute course, diffuse form, activity 1 with, skin lesions, sclerodactyly, ILD, RP, esophagitis, gastritis	16	2	2,67	1:640, AC-3, AC-21	CENP-B	6	IL6ST(chr5:55265588 AT/A)-LP<br>IL6ST(chr5:55265655 G/C)-VUS<br>SAMD9L(chr7:92761606 GT/G)-LP<br>ABCC2(chr10:101578956 CA/C)-P<br>JAZF1(chr7:28220153 C/T)-VUS
S29	f	SSc, chronic course, activity 1, skin lesions, RP, esophagitis, arthralgia, ILD	20	2	9,06	1:2560 AC-4/5	SS-A/52, U1-snRNP, RNP/Sm	31	SAMD9L(chr7:92761606 GT/G)-LP<br>ABCC2(chr10:101603641 CA/C)-LP<br>TNFAIP3(chr6:138199775 T/TC)-LP
