# Supplementary material for: Systemic Sclerosis in Kazakh Patients: A Preliminary Case–Control Immunogenetic Profiling Study
Source: Pathophysiology. 2025 Oct 28;32(4):57. doi: 10.3390/pathophysiology32040057 (PMC12641709; doi:10.3390/pathophysiology32040057)
Supplement: Supplementary file 1 [file pathophysiology-32-00057-s001.zip › Supplementary Table S2.pdf]

SUPPLEMENTARY MATERIAL

Systemic Sclerosis in Kazakh Patients: A Preliminary Case–-Control Immunogenetic Profiling Study

Lina Zaripova*, Abay Baigenzhin, Alyona Boltanova, Zhanna Zhabakova, Maxim Solomadin, Larissa Kozina

JSC National Scientific Medical Center, Astana, Kazakhstan

Supplementary Table S2. Clinical and laboratory characteristics of the individuals from control group. AC – anti-cell (ICAP, www.anapatterns.org); ANF – anti-nuclear factor; f – female; LP - likely pathogenic variant; m – male; P-pathogenic variant; VUS - variant of uncertain significance.

№	Gender	Chronic diseases	Allergy	Heredity	IL-6	ANF (<1:80, negative, AC-0)	Antibodies	Genetic variants
C01	f	no	no	not burdened	1.83	negative	not detected	LY96(chr8:74922341 CT/C)-VUS
C02	f	no	no	not burdened	0.67	negative	not detected	LY96(chr8:74922341 CT/C)-VUS<br>PTPN22(chr1:114381166 CT/C) –VUS<br>IRAK1(chrX:153278833 GCCCG/GCC)-VUS<br>REL(chr2:61149099 GT/G)-LP<br>SFTPD(chr10:81697984 A/G)-VUS
C04	f	no	no	not burdened	2.58	negative	not detected	LY96(chr8:74922341 CT/C)-VUS
C09	f	no	cold urticaria	rheumathoid arthritis (mother)	1.46	negative	not detected	PTPN22(chr1:114381166 CT/C) –VUS<br>SAMD9L(chr7:92764981 T/TT)-LP<br>PTPRC(chr1:198682102 AT/A)-LP
C10	f	chronic rhinitis	urticaria to cucumbers	not burdened	2.58	negative	not detected	PTPN22(chr1:114381166 CT/C) –VUS<br>SAMD9L(chr7:92761606 GT/G)-LP
C11	f	no	no	not burdened	0.86	negative	not detected	PTPN22(chr1:114381166 CT/C) –VUS<br>IL6ST(chr5:55247857 GT/G)-LP<br>IL6ST(chr5:55265588 AT/A)-LP

C13	m	no	no	not burdened	1.29	negative	not detected	PTPN22(chr1:114381166 CT/C) –VUS<br>CTLA4(chr2:204736165 G/GT)-LP
C15	f	no	no	not burdened	2.07	negative	not detected	PTPN22(chr1:114381166 CT/C) –VUS<br>PTPRC(chr1:198682102 AT/A)-LP
C16	f	no	no	not burdened	1.81	negative	not detected	SFTPD(chr10:81697984 A/G)-VUS<br>TNFAIP3(chr6:138199775 T/TC)-LP
C17	f	no	no	not burdened	2.41	negative	not detected	PTPN22(chr1:114381166 CT/C) –VUS
C18	f	no	no	not burdened	2.15	negative	not detected	PTPN22(chr1:114381166 CT/C) –VUS<br>IL6ST(chr5:55265655 G/C)-VUS
C22	f	no	no	not burdened	1.12	negative	not detected	PTPN22(chr1:114381166 CT/C) –VUS<br>SLC5A11(chr16:24918057 CA/C)-VUS
C23	f	no	no	not burdened	2.24	negative	not detected	PTPN22(chr1:114381166 CT/C) –VUS<br>REL(chr2:61149099 GT/G)-LP<br>PTPRC(chr1:198682102 AT/A)-LP
C24	f	no	no	not burdened	4.05	negative	not detected	SLC5A11(chr16:24918057 CA/C)-VUS
C25	f	no	no	not burdened	2.07	negative	not detected	PTPN22(chr1:114381166 CT/C) –VUS<br>SLC5A11(chr16:24918057 CA/C)-VUS<br>CTLA4(chr2:204736165 G/GT)-LP
C26	m	no	no	not burdened	2.07	negative	not detected	PTPN22(chr1:114381166 CT/C) –VUS<br>CTLA4(chr2:204732740 G/GT)-LP
C27	f	no	no	not burdened	3.28	negative	not detected	PTPN22(chr1:114381166 CT/C) –VUS<br>CTLA4(chr2:204732740 G/GT)-LP
C28	f	no	no	not burdened	4.85	negative	not detected	PTPN22(chr1:114381166 CT/C) –VUS<br>CTLA4(chr2:204732740 G/GT)-LP<br>BLK (chr8:11405631 AG/A)-VUS
