# Supplementary material for: Systemic Sclerosis in Kazakh Patients: A Preliminary Case–Control Immunogenetic Profiling Study
Source: Pathophysiology. 2025 Oct 28;32(4):57. doi: 10.3390/pathophysiology32040057 (PMC12641709; doi:10.3390/pathophysiology32040057)
Supplement: Supplementary file 1 [file pathophysiology-32-00057-s001.zip › Supplementary Table S3.pdf]

SUPPLEMENTARY MATERIAL

Systemic Sclerosis in Kazakh Patients: A Preliminary Case-Control Immunogenetic Profiling Study

Lina Zaripova*, Abay Baigenzhin, Alyona Boltanova, Zhanna Zhabakova, Maxim Solomadin, Larissa Kozina

JSC National Scientific Medical Center, Astana, Kazakhstan

Supplementary Table S3. Genetic variants description and population frequency databases. dbSNP - database of Single Nucleotide Polymorphisms; gnomAD - Genome Aggregation Database; LP - likely pathogenic variant; N/A - not available; P - pathogenic variant; SNV - Single Nucleotide Variant; TOPMed - Trans-Omics for Precision Medicine; VUS - variant of uncertain significance.

N	Gene	Locus	Genotype	Type	ACMG classify-cation	Description	Population frequency databases
1	SAMD9L	chr7:92764981	T/TT (reference sequencing TC)	INDEL SNV	LP	SAMD9L gene provides instructions for making a protein which is involved in regulating the growth and division (proliferation) and maturation/differentiation of cells, particularly cells in the bone marrow that give rise to blood cells.	gnomAD – N/A<br>1000 Genomes - N/A<br>dbSNP - N/A<br>TOPMed - N/A
		chr7:92761606	GT/G	INDEL	LP		gnomAD – N/A<br>1000 Genomes - N/A<br>dbSNP - N/A<br>TOPMed - N/A
2	LY96	chr8:74922341	CT/C	INDEL	VUS	This gene encodes a protein which associates with toll-like receptor 4 on the cell surface and confers responsiveness to lipopolysaccharide (LPS), thus providing a link between the receptor and LPS signalling.	gnomAD – N/A<br>1000 Genomes - N/A<br>dbSNP - N/A<br>TOPMed - N/A
3	REL	chr2:61149099	GT/G	INDEL	LP	This gene encodes a protein that belongs to the Rel homology domain/immunoglobulin-like fold, plexin, transcription factor	gnomAD – N/A<br>1000 Genomes - N/A<br>dbSNP - N/A<br>TOPMed - N/A

						(RHD/IPT) family. Members of this family regulate genes involved in apoptosis, inflammation, and oncogenic processes. This proto-oncogene plays a role in the survival and proliferation of B lymphocytes. REL is critical for T- and B-cell function in the immune system, as it binds to the promoters of genes that encode cytokines important for immunity against infectious pathogens, including IL2, IFNG, IL12 and IL23.	
4	IRAK1	chrX:153278833	GCC/GCCG	INDEL	VUS	This gene encodes the interleukin-1 receptor-associated kinase 1, one of two putative serine/threonine kinases that become associated with the interleukin-1 receptor (IL1R) upon stimulation. This gene is partially responsible for IL1-induced upregulation of the transcription factor NF-kappa B.	gnomAD – N/A<br>1000 Genomes - N/A<br>dbSNP - N/A<br>TOPMed - N/A
		chrX:153278833	GCCCG/GCC	INDEL	VUS		gnomAD – N/A<br>1000 Genomes - N/A<br>dbSNP - N/A<br>TOPMed - N/A
5	RBPJ	chr4:26417097	GTTTTTTGC/GTTTTTTTG ref GTTTTTTTGC	INDEL	VUS	The protein encoded by this gene is a transcriptional regulator important in the Notch signalling pathway. The encoded protein acts as a repressor when not bound to Notch proteins and an activator when bound to Notch proteins. It is thought to function by recruiting chromatin remodelling complexes containing histone deacetylase or histone	gnomAD – N/A<br>1000 Genomes - N/A<br>dbSNP - N/A<br>TOPMed - N/A
		chr4:26417097	GT/G	INDEL	VUS		gnomAD – N/A<br>1000 Genomes - N/A<br>dbSNP - N/A<br>TOPMed - N/A
		chr4:26426085	C/CT	INDEL	VUS		gnomAD – N/A<br>1000 Genomes - N/A

						acetylase proteins to Notch signalling pathway genes.	dbSNP - N/A<br>TOPMed - N/A
6	IL6ST	chr5:55265588	AT/A	INDEL	LP	The protein encoded by this gene is a signal transducer shared by many cytokines, including interleukin 6 (IL6), ciliary neurotrophic factor, leukaemia inhibitory factor, and oncostatin M. This protein functions as a part of the cytokine receptor complex. The activation of this protein is dependent upon the binding of cytokines to their receptors.	gnomAD – N/A<br>1000 Genomes - N/A<br>dbSNP - N/A<br>TOPMed - N/A
		chr5:55265655	G/C	SNV	VUS		0.0054%-gnomAD (Max)<br>0.0004%-gnomAD (Aggregated) East Asian<br>0.0289%-GenomeAsia (North East Asian)
7	TNFAIP3	chr6:138199775	T/TC	INDEL	LP	The TNFAIP3 gene encodes an ubiquitin-editing enzyme with a critical function in the inhibition of key proinflammatory molecules to negatively regulate inflammation and the immune response.	gnomAD – N/A<br>1000 Genomes - N/A<br>dbSNP - N/A<br>TOPMed - N/A
8	ITGA2	chr17:42453072	G/GC (ref GCC)	INDEL	LP	This gene encodes a member of the integrin alpha chain family of proteins. The encoded preproprotein is proteolytically processed to generate light and heavy chains that associate through disulfide linkages to form a subunit of the alpha-IIb/beta-3 integrin cell adhesion receptor.	gnomAD – N/A<br>1000 Genomes - N/A<br>dbSNP - N/A<br>TOPMed - N/A
		chr17:42455791	G/A	SNV	VUS		0.0363%-gnomAD (Max)<br>0.0132%-gnomAD (Aggregated): European-Non Finnish -0.0164%<br>Ashkenazi Jewish -0.0292%<br>South Asian -0.0363%<br>American -0.0028%<br>0.1875%-Iranome<br>0.1657%-Turkish Variome
9	ABCC2	chr10:101578956	CA/C	INDEL	P	ABCC2 is a 190-kD integral membrane glycoprotein expressed	gnomAD – N/A<br>1000 Genomes - N/A

						mainly in the canalicular (apical) membrane of liver cells. It belongs to the ATP-binding cassette transporter superfamily and transports endogenous and exogenous anionic conjugates from hepatocytes to bile.	dbSNP - N/A<br>TOPMed - N/A
		chr10:101603641	CA/C	INDEL	LP		gnomAD – N/A<br>1000 Genomes - N/A<br>dbSNP - N/A<br>TOPMed - N/A
		chr10:101559041	CA/C	INDEL	LP		gnomAD – N/A<br>1000 Genomes - N/A<br>dbSNP - N/A<br>TOPMed - N/A
10	AIRE	chr21:45708278	G/C	SNV	VUS	The AIRE gene encodes a transcriptional regulator that forms multimeric nuclear protein complexes in superenhancer chromatin regions within medullary thymic epithelial cells (mTECs), where it mediates the expression of tissue-specific antigens and facilitates immunologic self-tolerance.	gnomAD – N/A<br>1000 Genomes - N/A<br>dbSNP - N/A<br>TOPMed - N/A
		chr21:45711068	TC/T	INDEL	LP		gnomAD – N/A<br>1000 Genomes - N/A<br>dbSNP - N/A<br>TOPMed - N/A
		chr21:45713024	A/G	SNV	VUS		0.0325%-gnomAD (Max)<br>0.0197%-gnomAD (Aggregated): European-Non Finnish -0.0299%<br>South Asian -0.0231%<br>American -0.0235%<br>0.4469%-Turkish Variome<br>0.0629%-Iranome
		chr21:45711025	C/G	SNV	VUS		0.0294%-gnomAD (Max)<br>0.0111%-gnomAD (Aggregated): European-Non Finnish -0.0165%<br>African -0.004%<br>South Asian -0.0294%<br>0.1703%-Turkish Variome

							0.125%-Iranome<br>0.0602%-India DB
11	IL6R	chr1:154378136	GC/G	INDEL	LP	This gene encodes a subunit of the IL6 receptor complex. Interleukin 6 is a potent pleiotropic cytokine that regulates cell growth and differentiation and plays an important role in the immune response.	gnomAD – N/A<br>1000 Genomes - N/A<br>dbSNP - N/A<br>TOPMed - N/A
		chr1:154401686	G/A	SNV	VUS		0.012%-gnomAD (Max)<br>0.0021%-gnomAD (Aggregated): European-Non Finnish -0.0016%<br>African -0.012%<br>American -0.0028%<br>0.0504%- Greater Middle East Variome
12	JAZF1	chr7:28220153	C/T	SNV	VUS	This gene encodes a nuclear protein with three C2H2-type zinc fingers, and functions as a transcriptional repressor. Chromosomal aberrations involving this gene are associated with endometrial stromal tumours.	0.014%-gnomAD (Max)<br>0.0064%-gnomAD (Aggregated): European-Non Finnish -0.0126%<br>European-Finnish -0.004%
13	IKZF3	chr17:37922552	T/C	SNV	VUS	The IKZF3 gene encodes a transcription factor with an essential role in haematopoiesis.	0.1414%-gnomAD (Max)<br>0.0111%-gnomAD (Aggregated): European-Non Finnish -0.0009%<br>East Asian -0.1414%<br>South Asian -0.0033%<br>0.64%-4.7 Japanese
14	AFF3	chr2:100623846	GT/G	INDEL	LP	This gene encodes a tissue-restricted nuclear transcriptional activator that is preferentially expressed in lymphoid tissue.	gnomAD – N/A<br>1000 Genomes - N/A<br>dbSNP - N/A<br>TOPMed - N/A
15	TREX1	chr3:48508185	T/TC	INDEL	LP	The TREX1 gene provides instructions for making the three	gnomAD – N/A<br>1000 Genomes - N/A

						prime repair exonuclease 1 enzyme. This enzyme is a DNA exonuclease, which means that it trims molecules of DNA by removing DNA building nucleotides from the ends. Unusual pieces of DNA may be mistaken by cells for the genetic material of viral invaders, triggering immune system reactions. The three prime repair exonuclease 1 enzyme removes pieces of DNA that might otherwise set off an immune response.	dbSNP - N/A<br>TOPMed - N/A
16	IL18	chr11:112014401	C/T	SNV	VUS	The protein encoded by this gene is a proinflammatory cytokine of the IL-1 family that is constitutively found as a precursor within the cytoplasm of a variety of cells including macrophages and keratinocytes.	0.1004%-gnomAD (Max)<br>0.0081%-gnomAD (Aggregated): European-Non Finnish -0.0018%<br>East Asian -0.1004%<br>0.177%-Turkish Variome<br>0.0866%-GenomeAsia: North East Asian -0.4273%
17	IL12B	chr5:158749513	T/C	SNV	VUS	This gene encodes a subunit of interleukin 12, a cytokine that acts on T and natural killer cells and has a broad array of biological activities. IL12 is expressed by activated macrophages that serve as an essential inducer of Th1 cells development.	0.0381%-gnomAD (Max)<br>0.0028%-gnomAD (Aggregated): East Asian 0.0381%<br>0.09%-4.7 Japanese
18	PRKCQ	chr10:6527154	AT/A	INDEL	VUS	The protein encoded by this gene is one of the PKC family members. It is a calcium-independent and phospholipid-dependent protein	gnomAD – N/A<br>1000 Genomes - N/A<br>dbSNP - N/A<br>TOPMed - N/A

						kinase which is important for T-cell activation.	
19	PXK	chr3:58385095	T/C	SNV	VUS	This gene encodes a phox domain-containing protein which may be involved in synaptic transmission and the ligand-induced internalization and degradation of epidermal growth factors.	0.3259%-gnomAD (Max) 0.0237%-gnomAD (Aggregated): East Asian -0.3259% South Asian -0.0033% 0.16%-4.7 Japanese
20	DNASE1L3	chr3:58183626	G/T	SNV	VUS	Predicted to enable DNA binding activity and deoxyribonuclease I activity. Involved in apoptotic DNA fragmentation. Located in endoplasmic reticulum and nucleus.	0.0018%-gnomAD (Max) 0.0008%-gnomAD (Aggregated): European-Non Finnish 0.0018%
